# Drug Combinations: Mathematical Modeling and Networking Methods

**DOI:** 10.3390/pharmaceutics11050208

**Published:** 2019-05-02

**Authors:** Vahideh Vakil, Wade Trappe

**Affiliations:** Wireless Information Network Laboratory (WINLAB), Rutgers, The State University of New Jersey, New Brunswick, NJ 08901, USA; vavakil@winlab.rutgers.edu

**Keywords:** drug combinations, mathematical modeling, pharmacodynamics, network medicine, signaling network, evolutionary dynamics

## Abstract

Treatments consisting of mixtures of pharmacological agents have been shown to have superior effects to treatments involving single compounds. Given the vast amount of possible combinations involving multiple drugs and the restrictions in time and resources required to test all such combinations in vitro, mathematical methods are essential to model the interactive behavior of the drug mixture and the target, ultimately allowing one to better predict the outcome of the combination. In this review, we investigate various mathematical methods that model combination therapies. This survey includes the methods that focus on predicting the outcome of drug combinations with respect to synergism and antagonism, as well as the methods that explore the dynamics of combination therapy and its role in combating drug resistance. This comprehensive investigation of the mathematical methods includes models that employ pharmacodynamics equations, those that rely on signaling and how the underlying chemical networks are affected by the topological structure of the target proteins, and models that are based on stochastic models for evolutionary dynamics. Additionally, this article reviews computational methods including mathematical algorithms, machine learning, and search algorithms that can identify promising combinations of drug compounds. A description of existing data and software resources is provided that can support investigations in drug combination therapies. Finally, the article concludes with a summary of future directions for investigation by the research community.

## 1. Introduction

Biological systems are complex, with many interacting subsystems that operate at different functional levels. These different subsystems operate in an interconnected manner, with many complementary functions and redundancies in place to protect the organism from malfunction. Consequently, when a disease arises, the malady is typically a malfunction of multiple subsystems and thus is a complex phenomenon in its own right. As a result, the treatment of diseases should aim to address the multi-faceted challenges of the disease, rather than treating a single aspect of the disease’s cause, and thus often necessitates the combination of multiple drugs in a treatment.

While there may be benefits from using a single compound in the treatment of a disease, treatments consisting of mixtures of pharmaceutical agents often have superior effects to treatments involving single compounds. A well-known example of this arises in the treatment of migraines, in which drug cocktails are more effective than treatment with a single drug [1,2]. Acetaminophen (Paracetamol), aspirin (ASA), ibuprofen, and an aspirin-acetaminophen-caffeine combination (AAC) are common over-the-counter (OTC) products that are used by patients to reduce the symptoms of migraine headaches [2]. Although the Food and Drug Administration (FDA) has not formally approved the effectiveness of acetaminophen for migraine, it has been shown that 1000 mg of this drug can treat moderate pains [1]. Administering 900–1000 mg of aspirin provides an effective treatment for migraines, while 200–400 mg of ibuprofen is also effective [1]. In [3], through placebo-controlled studies, it was proven that the combination of acetaminophen, aspirin, and caffeine is highly effective for the treatment of migraine headaches. Furthermore, in [4], it was statistically shown that this cocktail of drugs is significantly superior to the application of a single drug, or even two compounds. This observation was further supported in another study [5].

The determination of drug dosages is important for obtaining optimum therapeutic results and for clinical tests in the drug development process [6]. Dosage algorithms aim to optimize the onset, intensity and duration of the therapeutic effects of the drugs by determining the appropriate dosages, frequency, and route of administration, while also minimizing any adverse effects. Due to the redundancy inherent in biological networks, it has been shown that the combination of multiple drugs can provide more effective responses than using a single drug, such as observed in cancer therapies [7,8,9]. Furthermore, the observation that multiple, synergistic drugs can be effective at smaller doses than single compounds highlights the importance of determining the proper doses in combination therapies. Hence, the design of algorithms that determine multi-drug dosages is important to ensuring effective treatments. However, given the vast amount of possible multi-drug combinations and the restrictions in time and resources needed to test all such combinations, optimizing drug dosage in combination therapies is a challenging issue. Further complicating dosage determination are issues of heterogeneity and resistance, which have been identified as some of the most important challenges facing cancer therapy, and require strategic drug combination methods [8].

Determining the proper dosages involving a combination of multiple pharmacologic agents is a complicated optimization problem that requires understanding the diminishing returns experienced as dosages increase beyond a minimal treatment level, the manner in which multiple agents combine to become more effective than a single agent, and the tradeoffs that exist between using multiple drugs. Mathematical models that capture these relationships are fundamental to determining drug dosage and schedules, and make it possible to arrive at suitable treatment plans by reducing the number of test cases involved in clinical trials. It is suggested in [10] that drug combination methods should follow a standard framework with a clear definition of what it means for drugs to interact. Their view is that such a framework should be general enough to cover all cases while also being flexible enough to be adapted to practical obstacles. Furthermore, the framework should not be dependent on knowledge of the mechanism of action as often this is not completely known. The authors ultimately conclude that a standard reference framework cannot be realized through one single model for all biomedical situations, and instead conclude that a collective set of appropriate models might be required for analyzing drug combinations. This conclusion highlights the importance of having a suite of mathematical tools, ranging from models to data analysis algorithms, that can support the design of multi-drug therapies.

This article surveys the mathematical models in the literature that have been proposed for capturing the benefits and tradeoffs that exist in multi-drug combination therapies, as well as data analysis algorithms for multi-drug therapies. In Section 2, we survey drug combination methods that use basic pharmacodynamics equations to formulate multiple-drug interactions. In this section, we explore interactive and non-interactive models that are based on the concentration-effect relationships between the agents. Section 3 moves further and explores combination therapy methods that capture the response of a target through the biochemical and signaling networks. These methods model the dynamical behavior of the signaling pathways in different layers, from the receptor to target protein, and through studying the biochemical interactions. When it comes to networks, the arrangement of the elements of the networks, which here are the receptors, proteins, ligands, and inhibitors play an important role in defining the interactions, and intermediate and final outcomes. In Section 3.2, we study methods that model drug interactions based on the network topologies associated with the underlying biological processes of a disease. In Section 4, we explore the mathematical modeling of the time-varying dynamics of drug combinations. Unlike Section 2 and Section 3, which study combination methods from the perspective of outcome analysis, Section 4.1 investigates modeling the time-varying protein concentration with respect to drug combinations. Section 4.2 explores the use of evolutionary dynamical modeling for modeling the impact of multiple drugs on treatment resistance. In Section 5, we provide a summary and comparison of the investigated models. Recognizing that mathematical modeling can face challenges as one expands the scale and scope of a treatment, such as considering very large disease networks or a vast amount of potential drugs and targets, we then examine computational algorithms that can analyze data to predict promising drug combinations. Such algorithms typically employ machine learning and statistical inference to arrive at their predictions, and we provide a summary of this branch of multi-drug methods in Section 6. Additionally, we provide a review of some helpful tools and software for studying drug combinations and interaction networks, as well as describe some of the data resources available, in Section 7. In Section 8, we conclude the paper by highlighting the importance of connecting mathematical models to personalizing patient treatments.

## 2. Drug Combinations Based on Concentration-Effect Models

A pharmacologic agent’s response is related to its concentration at the site of action. At the site of the action, receptor binding can be described by the law of mass action as

(1) $$C+R\underset{k_{b}}{\overset{k_{f}}{⇌}}RC,$$

where *C*, *R*, and RC represent the drug, receptor, and drug-receptor complex, respectively. At equilibrium, when the forward and backward rates are equal,

(2) $$\frac{\left[RC\right]}{{\left[R\right]}_{T}}=\frac{\left[C\right]}{k_{d}+\left[C\right]}\;\;,$$

where [] corresponds to concentration and ${\left[R\right]}_{T}$ is the total molar concentration of the receptors. $k_{d}=\frac{k_{b}}{k_{f}}$ is a dissociation constant and is defined as the concentration at which a drug binds to half of the total receptors [6]. Since the number of total receptors is limited, (2) implies that the binding process is a saturable, capacity-limited process, and a maximum effect can be observed when all the receptors are occupied. Considering the difficulty of measurements at the site of action and the capacity-limited specification of the response-concentration relationship, the empirical pharmacodynamic model, sigmoidal $E_{max}$, has been proposed

(3) $$E\left(c\right)=\frac{E_{max}c^{n}}{EC_{50}^{n}+c^{n}}\;\;,$$

where *c* is the concentration of the drug, and $EC_{50}$ is the concentration that produces half of the drug’s maximum response ($E_{max}$). This is a metric that defines the drug potency and depends on a drug’s affinity and intrinsic efficacy. $E_{max}$ is governed by multiple factors including a drug’s efficacy, the number of receptors and the way that stimulus is relayed to produce a biological response. Typically, dose-response curves follow a sigmoidal relationship, in which the response is negligible for a wide range of low doses, then the response starts to increase for a range of intermediate dose levels, and finally the response flattens out at its maximal effect for a wide range of high dose levels. The shape of the dose-response curve then follows a characteristic ‘S’ shape associated with sigmoidal functions. In this model, *n* is a hill-type factor governing the sigmoidicity of the response. This slope factor is usually a non-integer and is used to improve the fitting of the model to observed data [6].

There are several methods in the literature that model the interaction of drugs in combination based on the dose-effect relation of each component. In the following we categorize these models based on their basic assumptions and their distinctions.

### 2.1. Loewe Additivity Model

The mutual effect of drugs can be categorized as additivism, synergism or antagonism based on the form of their interaction in the combination [11]. However, the definition of the last two depends on the definition of additive behavior for the agents. In [11], Loewe introduced a representation for the dose-effect relationship of two drugs in a combination, based on the isobole, for defining the additive interaction. An isobole is a graph in Cartesian coordinates with the doses of two drugs as the two axes. The graph depicts a curve consisting of dose pairs that, when combined, achieve a specified level of effect. The isobole has been used as the theoretical basis for determining the expected effect of the combination of drug pairs [12]. Using this definition, Loewe specified the synergistic and antagonistic effects as the deviation from the strictly additive behavior. Moreover, he concluded that the interactive mechanisms of the components cannot be distinguished through bio-statistical methods. The isobole equation for additive behavior of two drugs with concentration $\left(c_{1},c_{2}\right)$ in the concentration, is in the form of [13]

(4) $$\frac{c_{1}}{C_{1}}+\frac{c_{2}}{C_{2}}=1,$$

where $C_{1}$ and $C_{2}$ are the concentrations of the agents that produce the same amount of effect as the combination, when they act as single drug. Equation (4) determines the locus of all combinations of these two drugs that produce no interaction [13].

When the agents show more effectiveness in combination than each individual, the one corresponding to the smaller amount is essential to the combination. Therefore, the isobole equation for synergism is defined as

(5) $$\frac{c_{1}}{C_{1}}+\frac{c_{2}}{C_{2}}<1.$$

In contrast, antagonism occurs when the combination of drugs produces a less effective response than each individual component, and thus the corresponding isobole equation is [11,13]

(6) $$\frac{c_{1}}{C_{1}}+\frac{c_{2}}{C_{2}}>1.$$

**Figure 1:**
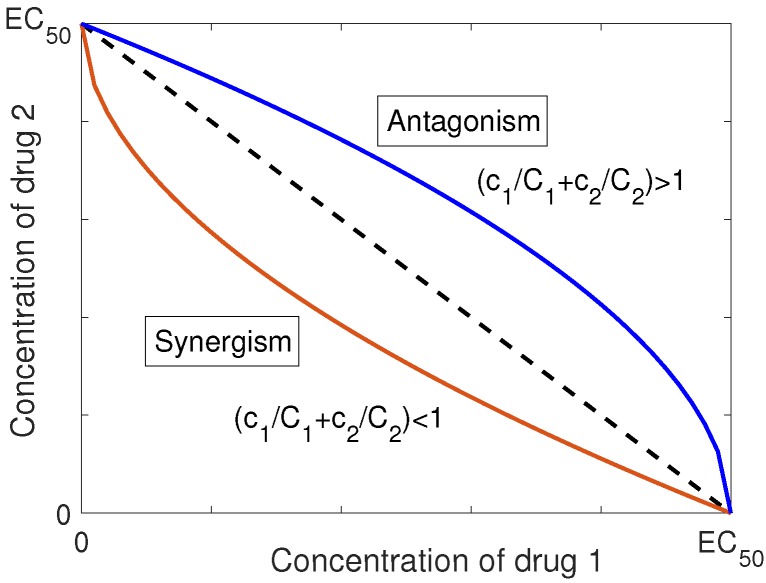
Isobole graph for synergism and antagonism, where the dash line represents the additivism.

Figure 1 represents the isobole graph for synergism and antagonism with respect to Loewe’s definition of additivism. The isobole method, which is a general approach to analyzing drug interaction, is an empirical method that is independent of the drugs’ dose-response relationship and the mechanism of interaction. It is worth noting that the isobole method does not necessarily show a consistent interaction for all possible doses. In other words, it could reflect synergism in one dosage regime, and antagonism in another regime, depending on the concentration of the agents in the combination [13].

#### 2.1.1. Combination of Non-Interactive Drugs

The isobole equation for *N* non-interactive agents, regardless of their dose-effect function, is in the form of

(7) $$\sum_{i=1}^{N}\frac{c_{i}}{C_{i}}=1,$$

where $c_{i}$ is the concentration of the $i^{th}$ drug in the compound, and $C_{i}$ is the equivalent concentration of each agent that produces the same level of effect when acting individually [14]. In other words, $C_{i}$’s are the concentrations at which

(8) $$\begin{array}{cc} E\left(C\right)=E_{i}\left(C_{i}\right), & for\;\;\;i=1,2,\cdots ,N. \end{array}$$

where *C* is the vector of the concentration of the agents, E(.) is the effect of the combination, and $E_{i}\left(.\right)$ represents the dose-effect relation of the $i^{th}$ drug, which in general could be any arbitrary monotonic function. Therefore, the following relationship is observed for every pair (i,j) of the agents in the combination

(9) $$C_{j}=E_{j}^{-1}\left(E_{i}\left(C_{i}\right)\right).$$

On the other hand, from (7), the equivalent concentration $C_{i}$ is determined as

(10) $$C_{i}=c_{i}{\left[1-\sum_{\overset{j=1}{i\neq j}}^{N}\frac{c_{j}}{C_{j}}\right]}^{-1}.$$

Regarding Equations (8)–(10), the effect of the combination drug can be computed as [14]

(11) $$E\left(C\right)=E_{i}\left(C_{i}\right)=E_{i}\left(c_{i}{\left[1-\sum_{\overset{j=1}{i\neq j}}^{N}\frac{c_{j}}{E_{j}^{-1}\left(E_{i}\left(C_{i}\right)\right)}\right]}^{-1}\right).$$

The authors in [13] provide a simpler expression for (11) when all the agents follow similar concentration-effect functions, i.e., every function can be expressed as a scaled version of another one:

(12) $$E\left(C\right)=E_{i}\left(c_{i}+\sum_{\overset{j=1}{i\neq j}}^{N}\frac{C_{i}}{C_{j}}\;c_{j}\right).$$

**Figure 2:**
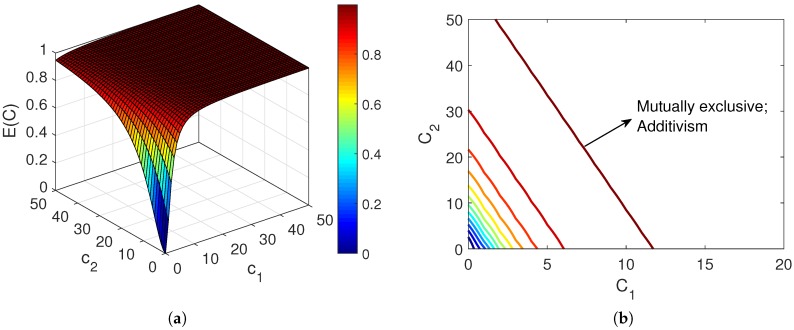
Combination of mutually exclusive agents: (**a**) The effect versus dosage of the combining agents; (**b**) Dosage of drug 2 versus drug 1 with respect to the combination effect which represents additivism.

- **Special case I: summation of the effects**
  In [14], it is proved, for non-interactive components, that their response $E_{i}\left(c_{i}\right)$ is a linear function of $c_{i}$, and the combination effect can be modeled by the summation of all the individual effects:
  (13) $$E\left(C\right)=\sum_{i=1}^{N}E_{i}\left(c_{i}\right),$$
  In the case of the non-linear relationship, the model can be applied when the concentration is small, and the non-linearity is negligible [14]. The proof has been shown using the isobole equation, Equation (7), for *N* non-interactive agents.
- **Special case II: multiplication of the effects**
  In [14], it was shown that if the combination effect of multiple drugs follows the multiplicative analog to Equation (7) as
  (14) $$E\left(C\right)=\prod_{i=1}^{N}E_{i}\left(c_{i}\right),$$
  then the individual concentration-effect relationships for the non-interacting drugs must follow an exponential model.
- **Special case III: mutually exclusive agents with sigmoidal concentration-response relationship**
  For interactions that follow the law of mass action (1), the concentration-response relationship is a sigmoidal function of the form of (3). Chou and Talalay showed that relationships that are governed by law of mass action can be represented by the generalized Median-Effect equation. They defined the fractional affected and fractional unaffected functions to describe the dose-effect relationship [15,16,17]. For a drug with concentration $c_{i}$, the fractional affected ($E_{i}\left(c_{i}\right)$) and fractional unaffected ($U_{i}\left(c_{i}\right)$) can be represented generally as
  (15a) $$\begin{array}{cc} E_{i}\left(c_{i}\right) & =\frac{c_{i}^{n_{i}}}{M_{i}^{n_{i}}+c_{i}^{n_{i}}}, \end{array}$$
  (15b) $$\begin{array}{cc} U_{i}\left(c_{i}\right) & =\frac{M_{i}^{n_{i}}}{M_{i}^{n_{i}}+c_{i}^{n_{i}}}, \end{array}$$
  where $M_{i}$ is the median effective concentration of the $i^{th}agent$, which can be replaced by its $EC_{50}$, and $n_{i}$ determines the sigmoidicity of the corresponding agent, which is related to the number of binding sites [13]. Therefore, in the case of mutually exclusive agents that share binding sites, all agents should have equal sigmoidicity, i.e., $n_{i}=n$ for all i=1,⋯,N, and thus they all follow a similar concentration-response function. Accordingly, Equation (12) can be applied to predict the affected and unaffected responses of the combination [13], and thereby derive Chou and Talalay’s combination formula for mutually exclusive agents [15]:
  (16) $${\left(\frac{E\left(C\right)}{U\left(C\right)}\right)}^{1/n}=\sum_{i=1}^{N}\frac{c_{i}}{M_{i}}=\sum_{i=1}^{N}{\left(\frac{E_{i}\left(c_{i}\right)}{U_{i}\left(c_{i}\right)}\right)}^{1/n}.$$
  In the above equation, it should be noted that in this situation $\frac{C_{i}}{C_{j}}=\frac{M_{i}}{Mj}$ [13]. Figure 2 shows the combination effect of two mutually exclusive drugs, which confirms their additive relationship.
  It is worth noting that considering the definition of the fractional affected and unaffected functions, E(c)+U(c)=1, for any individual or combined agent. In the case of first-order agents, i.e., n=1, Equation (16) reduces to
  (17) $$\frac{1}{U\left(C\right)}=\sum_{i=1}^{N}\frac{1}{U_{i}\left(c_{i}\right)}-\left(N-1\right).$$
  Then the fractional affected response can be found directly as $E\left(C\right)=1-U\left(C\right)$. Expression (17) confirms the equation introduced by Chou and Talalay in [17] for first-order inhibitors that follow Michaelis-Menten kinetics.
  Unfortunately, the ideal case involving similar sigmoidicity of the combining agents is not the case that is commonly observed in practice. Considering two drugs (i,j) with $\left(n_{i},n_{j}\right)$, the solution presented in [13] involves substituting
  (18) $$E_{j}^{-1}\left(E_{i}\left(C_{i}\right)\right)=M_{j}{\left(\frac{C_{i}}{M_{i}}\right)}^{n_{i}/n_{j}}$$
  into (9) and then calculating $C_{i}$ iteratively using (10) through
  (19) $$C_{i}\left(1-\frac{c_{j}}{Mj}{\left(\frac{M_{i}}{C_{i}}\right)}^{n_{i}/n_{j}}\right)-c_{i}=0.$$
  The expected effect of the combination then can be calculated using the fact that $E\left(C\right)=E_{i}\left(C_{i}\right)$.

#### 2.1.2. Combination of Interacting Drugs

Berenbaum [13] defines interaction as the deviation from the expected response based on the concentration-effect relation of the components and regardless of their mechanism of action.

**Figure 3:**
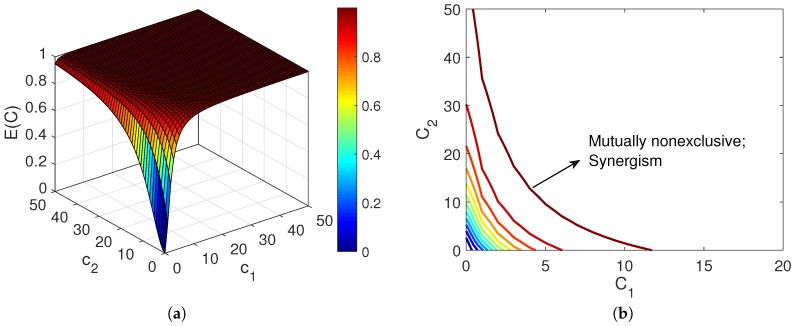
Combination of mutually nonexclusive agents: (**a**) The effect versus the concentration of the combining drugs; (**b**) Dosage of drug 2 versus drug 1 with respect to the combination effect which represents synergism.

- **Mutually nonexclusive agents with sigmoidal concentration-response relationship**
  It has been shown in [17,18], through kinetic analysis, that the effect of combining mutually nonexclusive inhibitors adhering to Michaelis-Menten kinetics, is synergism [13]. Chou and Talalay [15] showed that for the combination of two mutually nonexclusive agents (i,j) with equal sigmoidicity factor *n*, the ratio of affected and unaffected responses is expressed in the form of
  (20) $${\left(\frac{E\left(C\right)}{U\left(C\right)}\right)}^{1/n}=\frac{c_{i}}{M_{i}}+\frac{c_{j}}{M_{j}}+\frac{c_{i}\;c_{j}}{M_{i}\;M_{j}}.$$
  In comparison with (16), the last term in (20) is a factor that reflects the synergistic relationship between the agents [19]. However, Chou argued that since exclusiveness/non-exclusiveness could be present partially, the above equation may underrate the synergism. Hence, he suggested using (16) as the basic equation for evaluating the combination effect, and adding a “contributing factor” that reflects the synergism [19]. Figure 3 shows the combination effect of two mutually nonexclusive agents which represents their synergistic relationship.

#### 2.1.3. Extensions Based on Loewe’s Model

The authors in [20] propose a two-component model involving a parametric function that predicts the additive effect of two combining agents based on Loewe’s method, and a non-parametric function that represents the deviation from additivity and captures the synergistic and antagonistic interaction of the agents. The authors then estimate this function by minimizing the penalized least squares error between experimental results and the predicted Loewe additive effect. Lee et al. in [21,22] discuss a procedure to estimate a confidence interval for the interaction index in Loewe’s model by calculating a bound on the curve of interaction indices versus effects. Based on simulation and case studies, in [21], an analysis is presented for two-drug and multiple-drug combination scenarios. The authors conclude that their approach to finding the confidence bound is as effective as other confidence bound calculations via Monte Carlo techniques, but note that their approach has the benefit of being faster to compute. The authors in [23] present a two-stage response surface method based on Loewe’s model for improving the accuracy of synergy estimation by maximizing the use of data from single and combination therapies. In this method, the parameters corresponding to the effect of a single drug are estimated first, and then as a second stage the interaction index is estimated. The authors propose the use of Non-linear Least Squares (NLS) to estimate the parameter for the single-drug dose-response function [24]. They propose a quadratic model to estimate the interaction index subject to the estimation of dose-response parameters for single drugs. In [22] a method is presented for calculating the interaction index and its confidence interval based on the median-effect equation. Through experimental comparison they suggest that a general approach is required for estimating the interaction indices to capture the various scenarios where additivism, synergism and antagonism occur and, in particular, when there is an inconsistency in the interactive effects depending on the dosage of combining drugs. Moreover, they conclude that the design of an experiment should be performed in a manner that would allow one to capture all these variations.

### 2.2. Bliss Independence Model

Another classical approach to modeling the interaction between different agents is Bliss’s independence model. Bliss [25] defines three basic types of interactions for drug combinations based on the relative fractional affected concentration response of the agents. Broadly speaking, he classifies the joint action categories as: (i) independent, (ii) similar, and (iii) synergistic.

#### 2.2.1. Independent Joint Action

When the drugs being combined have dissimilar modes of action and act independently, the effect of the combination can be derived using the definition of independence from probability and statistics [13]. Bliss asserts that this type of action is the simplest case, in which the response can be predicted from the concentration response of each individual component and their “susceptibility correlation”, irrespective of their concentration ratio [25]. The combined effect of two agents (i,j), when *i* is more potent than *j*, in terms of the fractional affected response is formulated as

(21) $$E\left(C\right)=E_{i}\left(c_{i}\right)+E_{j}\left(c_{j}\right)\left(1-E_{i}\left(c_{i}\right)\right)\left(1-r\right),$$

where *r* represents the correlation between the susceptibility to the agents (i,j) and can be computed empirically. There are two extreme cases worth exploring, which correspond to when r=1 and r=0. The first case occurs when drug *i* is the dominant agent and the effect of the combination is the same as the effect of *i* when administrated alone, and thus $E\left(C\right)=E_{i}\left(c_{i}\right)$ [25]. The latter happens when there is no correlation between the agents and so (21) reduces to the well-known Bliss independence formula:

(22) $$E\left(C\right)=E_{i}\left(c_{i}\right)+E_{j}\left(c_{j}\right)-E_{i}\left(c_{i}\right)E_{j}\left(c_{j}\right).$$

This formula has an equivalent form in terms of fractional unaffected response:

(23) $$U\left(C\right)=U_{i}\left(c_{i}\right)U_{j}\left(c_{j}\right),$$

which is similar to the fractional product method of Webb [26]. The extension of the Bliss independence model to the case of *N* drugs is found using (23) and given as Equation (24) in the literature [27,28]:

(24) $$U\left(C\right)=\prod_{i=1}^{N}U_{i}\left(c_{i}\right).$$

**Figure 4:**
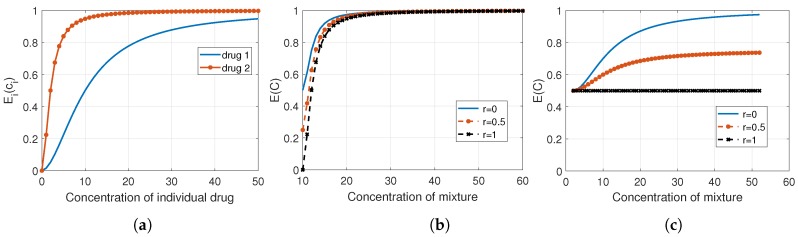
The combination effects of Bliss’s model; (**a**) The response of two combining agents; (**b**) The combination effect for different values of the correlation factor *r*, when the concentration of drug 1 is fixed at its $EC_{50}$ value while the concentration of drug 2 is varied; (**c**) The combination effect for different values of the correlation factor, when the concentration of drug 1 is varied while the concentration of drug 2 is fixed at its $EC_{50}$ value.

Figure 4 represents the effect of combining two drugs based on Bliss’s model. Figure 4a shows that drug 2 is more potent than 1, thus it governs the combination effect in (21) when r>0. Figure 4b,c present the combination response when the drugs are assumed independent (r=0), correlated (with r=0.5), and fully correlated (r=1). Since drug 2 is the most potent of these two combining agents, the distinction between these cases is not very clear in (b) where the concentration of drug 2 is changing while the concentration of drug 1 is kept fixed at its $EC_{50}$ value. The concentration of the mixture equals the total concentrations of drugs 1 and 2. However, the difference is obvious in (c), where the concentration of drug 2 is fixed at its $EC_{50}$ value while the concentration of drug 1 varies. It is apparent that in the case of full correlation (r=1), the combination effect is defined by the response of drug 2, which is $E_{2}\left(EC_{50}\right)$ in this example.

#### 2.2.2. Similar Joint Action

When two agents have similar effects, interact independently, and each one can be replaced by a specific proportion of the other one in the mixture without introducing any change in the combination response, the interaction is categorized by Bliss [25] as similar joint action. The response-concentration relation of the combination of drugs (i,j) in this group is the same as drug *i*, when calculated as

(25) $$E\left(C\right)=E_{i}\left(\frac{c_{i}}{\rho +k(1-\rho )}\right),$$

where ρ is the proportion of drug *i* in the combination and $k=\frac{E_{i}\left(c_{i}\right)}{E_{j}\left(c_{j}\right)}$ when *i* and *j* act individually.

#### 2.2.3. Synergistic Joint Action

Synergism (Antagonism) is defined by Bliss as a type of interaction in which the effect of the combination is more (less) potent than what is predicted from the individual agents [25]. Unlike the case of independent interaction, where the effect of the mixture is not related to the ratio of the concentration of the combining agents, in synergistic (antagonistic) interactions, the response of the combination is related to the ratio of the concentration of each agent to concentrations of the other components.

**Figure 5:**
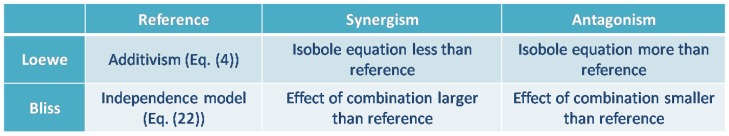
Comparison between Loewe and Bliss models’ definition of interaction.

Figure 5 compares Loewe’s and Bliss’s definitions of synergism and antagonism. In [29], the authors compare Loewe’s additivity and Bliss’s independence models when applying them to understand experimental results. They show that for some cases the two models are dramatically different, and that this can depend on the sigmoidicity of the dose-response functions. Notably, they found that when the hill factor governing the sigmoidicity is high, the Bliss independence model overestimates synergism, while the Loewe additivity model overemphasizes antagonism. The authors then conclude that for each case of multiple agents, the appropriate model should be determined by studying the experimental data. They propose that multiple experiments be performed in which agents are introduced synchronously and asynchronously. For example, considering in vitro experiments in which drug A and drug B administered at the same time, drug A is administered first and then subsequently drug B is administered, and drug B is administered first followed by drug A makes it possible to better hypotheses about the mechanisms of interaction and the appropriate combination model that matches the in vitro experiments.

#### 2.2.4. Extensions of the Bliss Independence Model

Recently, the authors of [27,28] have modified Bliss’s formula and proposed new experimentally validated models for predicting the effect of combining multiple anti-cancer drugs. The model, presented in [27], predicts the effect of a combination of three or more drugs based on the response of single drugs and mixtures of two compounds:

(26a) $$\begin{array}{c} U\left(C\right)=\prod_{i=1}^{N}U_{i}\left(C_{i}^{\left(e\right)}\right), \end{array}$$

(26b) $$\begin{array}{c} C_{i}^{\left(e\right)}=c_{i}\prod_{j=1,j\neq i}^{N}{\left(1+\alpha_{ij}\frac{C_{j}^{\left(e\right)}/M_{j}}{1+C_{j}^{\left(e\right)}/M_{j}}\right)}^{-1}, \end{array}$$

where $\alpha_{ij}$ is the interaction parameter, which is estimated using experimental data for the drug pair (i,j). Then, starting with $C_{1}^{\left(e\right)}=c_{1}$, the proposed method [27] numerically calculates $C_{i}^{\left(e\right)}$ for i>1. The model is validated through assaying 3 anti-cancer drugs at 8 doses for each drug. The proposed model does not require any information about the drugs’ mechanism of action. It does, however, require measuring the pairwise drug responses at multiple dosage levels. In [28], the authors propose a modified Bliss-based model that uses a single dose measurement for pairs of agents to predict the effect of a multiple-drug mixture. The model, which is called the pairs model, predicts the response of an *N*-drug mixture via:

(27) $$U\left(C\right)={\left(\prod_{\forall i,j}U_{ij}\left(C_{ij}\right)\right)}^{1/(N-1)},$$

where $U_{ij}\left(C_{ij}\right)$ follows the Bliss formula (23) for (i,j), and $C_{ij}$ is the concentration of the mixture. The authors in [28] validated the model using the combination of 6 anti-cancer drugs assayed on two cell lines. However, the restriction of the pairs model is that it can only predict the effect of the combination at the same doses that the pairwise measurements were performed at [28].

Except for the summation model for a specific case of non-interactive drugs, the methods presented in this section generally combine multiple drugs in a non-linear fashion. In Section 4.1, we explore a linear modeling of multiple drugs that has proven effective in capturing protein concentration responses to the combination of multiple drugs.

Beyond capturing pharmacodynamic aspects of multiple drugs being applied in pharmaceutical treatments, Bliss independence, and Loewe additivity have been applied to model population aspects of a treatment. In [30], the authors examine the application of multiple antimicrobial peptides (AMPs) to controlling the rate of growth in a bacterial population since the rate of growth for a pathogen population declines in a sigmoidal manner relative to the AMP concentration. The authors examine whether multiple antimicrobial agents have synergistic or antagonistic effects. By applying a multi-hit population model that reflects the dynamics of AMPs hitting target receptors on bacterial cells, the authors are able to show that the Bliss independence model is suitable when there is no interaction between the AMPs, while Loewe additivity is appropriate when the AMPs affect the same targets of a cell. We will further explore the mathematical modeling of dynamical aspects related to drug treatments in Section 4.

## 3. Drug Combinations Based on Network Analysis

Network medicine involves applying a network-centric approach to understanding the principles that govern the complex intercellular and intracellular interactions and molecular relationships underlying organism function and dysfunction [31]. In this regards, human molecular networks are broadly characterized as protein interaction networks, metabolic networks, regulatory networks and RNA networks [31]. In this approach, a disease is considered to be the result of a complex interaction among network elements, and potentially across network types. Consequently, network pharmacology, looks at the connectivity, redundancy, interaction, and the relationships between network nodes to design more efficient, less toxic drugs and treatments [32].

The authors in [33] use knowledge of a disease’s network to propose a drug-disease proximity metric. They employ this measure to quantify the interaction between a drug target and the disease to reveal the therapeutic effect of a drug. They conclude that a drug with a target proximal to a disease is more likely to be effective than a drug with a distant target. They also suggest that among unknown drugs, the ones proximal to disease targets are more likely to be clinically tested. However, they mention that there are limitations to network-based drug-disease proximity analysis, which are largely the result of the incomplete knowledge of the interaction network. In [34] the authors suggest that the geometric position of disease modules is related to their functionality and presents a geometric dissimilarity metric to classify disease modules with similar clinical characteristics. While the proximity metric proposed in [33] is shown to be successful for the case of a single drug-disease module, in [35] Cheng et al. apply a different measure, introduced in [36], for quantifying the network-based relationship between two drugs and their targets and provide a network approach to determining effective drug combinations. The so-called separation measure shows whether the targets of the two drugs are in the same network vicinity or whether they are topologically separated, and this is related to their chemical and functional similarities. They suggest six topologically different classes for two drug-target and drug-disease modules based on their potential overlaps. Through a statistical investigation of drugs for hypertension, they find that only the class of separated drug-target modules that have individual overlap with the drug-disease module shows significant effectiveness over single-drug therapy.

Improved drug combinations can be realized by identifying multiple targets and pathways that are systematically involved in the cellular dysfunction associated with a disease [31]. This field can also profit from identifying a combination of nodes in the molecular network whose interaction leads to a desired therapeutic result [32]. We explore such approaches in the following subsections by studying combination methods based on signaling networks, and more generally combinations based on the topology of the network.

### 3.1. Signaling and Biochemical Networks

Exploring the response of an activated protein to an inhibitor in different biological and biochemical situations is a fundamental step in the drug development process. Compounds that act as inhibitors, however, react with their targets through signaling pathways that are part of a complex signaling network. Conventional methodologies, which are based on dose-effect models, do not reflect the dynamical behavior of the signaling network. Therefore, to provide a more precise understanding of drug effects it is beneficial to consider a networking approach and investigate the inhibitor interactions within biochemical and signaling networks.

One of the earliest works in modeling and simulating signaling networks to predict the effects of drug combinations was performed by Jackson [37]. Jackson presented a mathematical model that uses a system of ordinary differential equations (ODE) involving 63 rate equations representing chemical reactions within the biochemical pathways of folate, and the nucleotide metabolism associated with DNA biosynthesis. Jackson simulated his model to predict the interaction between multiple pairs of anti-cancer drugs applied to two different cell lines, and reports that the simulation results are in good agreement with the experimental results in terms of predicting synergism and antagonism effects. Jackson shows that his network model can explain the experimental results based on the structure of the pathway and the mechanism by which the inhibitors interact with the substrates. Thus, the network model leads to a more accurate prediction of the combination effect. However, he admits that representing a complex network with a simplified model involves approximations in the chemical rate equations and in numerical calculations, which are new sources of error that this method might have to face.

**Figure 6:**
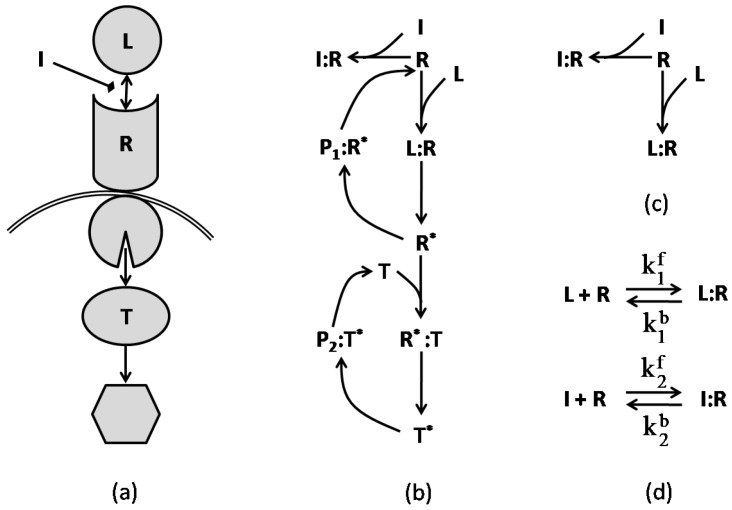
Single target pathway, where L is the ligand, R is the receptor, T is the target protein, *I* is the inhibitor, $R^{*},T^{*}$ are activated receptor and protein, and $P_{1},P_{2}$ are phosphotase: (**a**) Regulatory graph; (**b**) Reaction graph; (**c**) Basic reaction graph involving the ligand, inhibitor and receptor; (**d**) Chemical reaction network for the basic graph.

**Figure 7:**
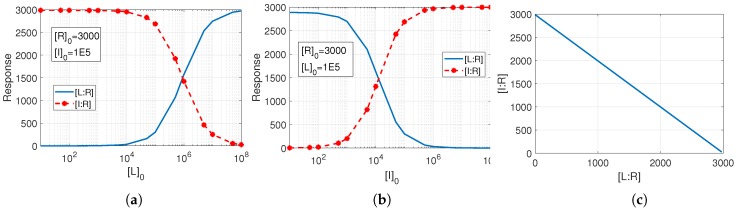
The steady-state response of (29): (**a**) versus the initial concentration of the ligand when the inhibitor’s initial concentration is constant; (**b**) versus the inhibitor’s initial concentration when the initial concentration of the ligand is fixed; and (**c**) the receptor’s response to the inhibitor versus its response to the ligand.

In [38], the authors present multiple simplified models of typical networks that model combination therapies in signaling networks. Before exploring these models, we examine the network graphs of a simple pathway with a single inhibiting agent. Following the examples in [38], Figure 6 illustrates the regulatory and reaction schemes of a simple pathway. The target protein responds to the ligand and inhibitor in a manner related to its concentration and the concentrations of ligand, receptor, and inhibitor as well as the association/disassociation constants for all the underlying reactions. The figure also shows a basic reaction graph that includes the immediate receptor, ligand and inhibitor, and its corresponding chemical reaction network. The rate for each reaction is governed by the law of mass action, which can be calculated for the reactions of Figure 6d:

(28a) $$\begin{array}{cc} V_{L} & =k_{1}^{f}\left[L\right]\left[R\right]-k_{1}^{b}\left[L:R\right], \end{array}$$

(28b) $$\begin{array}{cc} V_{I} & =k_{2}^{f}\left[I\right]\left[R\right]-k_{2}^{b}\left[I:R\right], \end{array}$$

where [] defines the concentration of each species, and $k_{i}^{f},k_{i}^{b}$ are the reaction rates of the forward and backward equations, respectively, for i=1,2. The ODE system that models the dynamic behavior of this network is given in (29) for $C_{lr}=\left[L:R\right]$ and $C_{ir}=\left[I:R\right]$.

(29a) $$\begin{array}{cc} \frac{dC_{lr}}{dt} & =C_{lr}^{2}+C_{lr}C_{ir}-\left({\left[L\right]}_{0}+{\left[R\right]}_{0}+1/k_{a1}\right)C_{lr}-{\left[L\right]}_{0}C_{ir}+{\left[L\right]}_{0}{\left[R\right]}_{0}, \end{array}$$

(29b) $$\begin{array}{cc} \frac{dC_{ir}}{dt} & =C_{ir}^{2}+C_{lr}C_{ir}-\left({\left[I\right]}_{0}+{\left[R\right]}_{0}+1/k_{a2}\right)C_{ir}-{\left[I\right]}_{0}C_{lr}+{\left[I\right]}_{0}{\left[R\right]}_{0}, \end{array}$$

where ${\left[\;\right]}_{0}$ represents the initial concentration of the species, and $k_{ai}=k_{i}^{f}/k_{i}^{b}$ for i=1,2, is the affinity constant of the reversible reaction *i*. The steady-state solution of this system is represented in Figure 7 for two cases in which (a) the initial value of inhibitor is kept fixed and (b) the initial value of the ligand is constant. The figure illustrates the response of the receptor to variations of the ligand and inhibitor concentrations. It is apparent from the graphs that, while increasing the concentration of one agent raises the receptor’s response to that agent, it reduces the receptor’s response to the other agent. In other words, the ligand and inhibitor compete for the receptor, as specifically depicted in Figure 7c.

**Figure 8:**
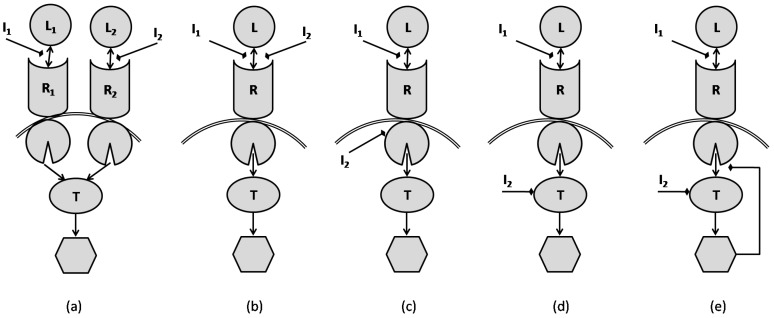
Drug combinations in signaling networks according to [38]: (**a**) Inhibition at two parallel pathways converging to a single target protein; (**b**) Inhibition at the same site of action; (**c**) Inhibition at different sites of action; (**d**) Inhibition at different levels; (**e**) Inhibition at different levels with a negative feedback loop.

Figure 8 shows different network models that are typical for inhibitor combinations as were presented in [38]. The inhibitors can be combined in many different ways, such as in a manner that inhibits the target through parallel pathways converging to a single common target (Figure 8a); or in the same pathway and at the same site of the receptor (Figure 8b); or at different sites of the receptor (Figure 8c). The inhibition could also happen at different levels of a pathway (Figure 8d) and this could be accompanied by a negative feedback from the activated target (Figure 8e). One interesting observation is that there is not a direct relationship between a simple signaling network and the classical notions of synergism and antagonism. For example, although one might want to classify a signaling scenario according to Loewe’s model, what one finds is that the relationship can be quite dependent on the underlying scenario and the parameters associated with the reaction equations. For the specific parameters used in the simulations of [38], it was shown that the combination of the inhibitors in Figure 8a, leads to synergism. The mechanism presented in the network of Figure 8b results in additivism, while the model of Figure 8c produces synergism. The effect of combination as in Figure 8d is additivism when single phosphorylation is involved, yet is synergism when double phosphorylation is involved [38]. However, the authors show that the negative feedback in Figure 8e makes double phosphorylation ineffective and leads to additivism.

In [39] using a mathematical model for the Epidermal Growth Factor Receptor (EGFR) signaling network, Araujo et al. show that the simultaneous inhibition of multiple nodes in the network produces synergism when compared with the targeting of a single node in the network. In addition, the authors suggest that in the case of targeting serially connected downstream signals, the synergistic effect is the result of the non-linear relationship between the kinetic parameters. Moreover, it is shown that the desired level of inhibition is achieved for lower doses of the combining agents when targeting multiple nodes of a signaling network than when compared to the required dose when attempting a single node inhibition.

### 3.2. Network Topology

Although the current state of knowledge associated with signaling networks is far from complete, it is nevertheless possible to capture much of the dynamics and tradeoffs that exist by recognizing that the networks associated with biological processes are generally built using smaller, recurrent networks, known as network motifs. These small networks, which occur frequently due to their evolutionary advantages, can be parameterized with a moderately sized parameter space that provides the flexibility to capture the dynamics of real biological networks, and thereby serves as an ideal starting point for exploring the design of multi-drug therapies that reflect therapeutic and toxic effects broadly.

**Figure 9:**
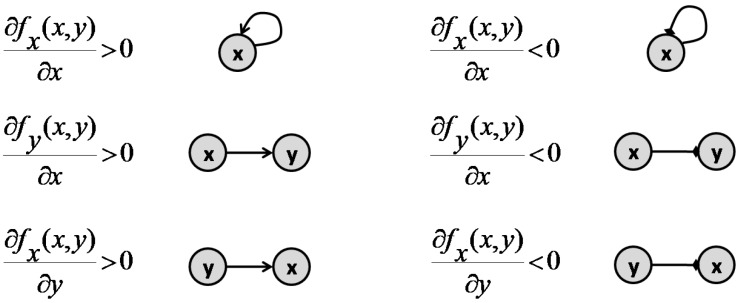
Representations for the basic interactions between the nodes in a two-element motif.

Consider a basic motif containing two nodes *x* and *y*, which represent two interacting species of the pathway or chemical reaction network. The interaction between these nodes are defined based on the ODE system that represents the reactions. Assuming a general ODE system in the form of

(30a) $$\begin{array}{cc} \frac{dx}{dt} & =f_{x}\left(x,y\right), \end{array}$$

(30b) $$\begin{array}{cc} \frac{dy}{dt} & =f_{y}\left(x,y\right), \end{array}$$

the sign of the entries of the Jacobian matrix of (30) specify the type of interaction between *x* and *y*. If $\frac{\partial f_{x}\left(x,y\right)}{\partial y}>0$, then *y* activates *x*, while if $\frac{\partial f_{x}\left(x,y\right)}{\partial y}<0$, *y* inhibits *x* [40]. If any of the partial derivatives is zero, there would be no connection in the corresponding direction. Figure 9 presents all the variations possible between *x* and *y*, as well as the graph representation for each interaction.

**Figure 10:**
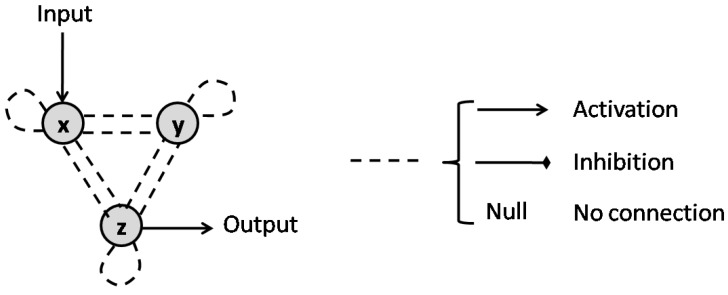
General representation of a three-node network where the dashed lines can be replaced by an arrow for activation or a blockage for inhibition. There would be no connection for no interaction.

**Figure 11:**
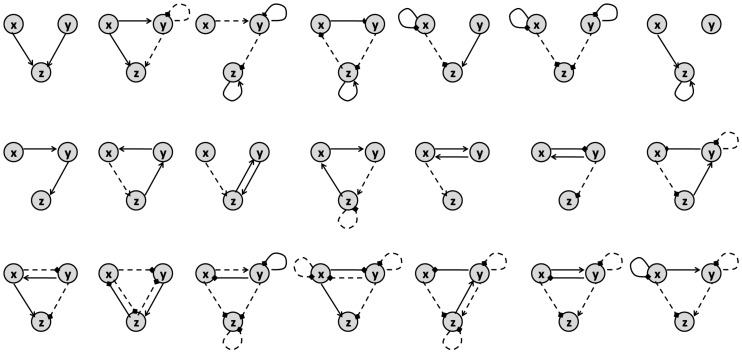
Set of the motifs that according to [41] leads to synergism for the combination of two drugs. Solid lines represent the drugs target links.

**Figure 12:**
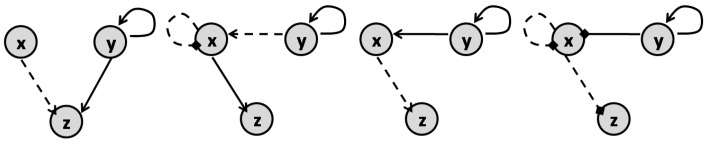
Set of the motifs that according to [41] leads to antagonism for a 2-drug mixture. Solid lines represent the target links of the drugs.

The authors of [41] study all variations of the motifs that model three-node enzymatic networks to show that synergism and antagonism of a combination can be defined by the topology of their target network. The general form for the motifs that they explore is represented in Figure 10. Their underlying enzymatic network was first investigated in [42] as a minimal network including one node as the receptor, one node as the output and one with any regulatory role. There are 16,038 possible variations of the 3-node motifs that have connections from the receiver node to the output node [41,42]. Yin et al. in [41] formulate the ODE system for all these motifs to find the steady-state solution of the output node’s concentration. They used these results as a metric of a drug’s efficacy when applied to these different motifs. After identifying the links in each motif that are targets of the inhibitors, they identified the conditions needed to achieve synergism and antagonism effects according to Loewe’s definition ((5) and (6)). They conclude that synergistic and antagonistic effects of drug combinations are largely dependent on the network topology of the target. They claim that the number of motifs that offer synergism is much more than those that show antagonism. Accordingly, Ref. [41] provides a list of 21 motifs that can lead to synergism and 4 motifs that can result in antagonism, as represented in Figure 11 and Figure 12, respectively.

In network-targeted combination therapy, the selection of target nodes is important in achieving an effective result and [43] suggests that the topology of the network plays a significant role in this choice. For example, using a mathematical model for a minimal six-node cell signaling network, the authors claim that in the case of a negative feedback loop, targeting the feedback’s output and input nodes leads to the most efficient therapy. On the other hand, in the case of a positive feedback loop, the node that is immediately connected to the feedback output node, in its downstream, is the best target.

Ultimately, the use of network models, when combined with knowledge of the underlying parameters and chemical reactions, allows one to have a well-described model with which to explore the behavior of a set of nodes in relation to each other. Such models are powerful in that they allow for the investigation of the effects of introducing one or more agents at specific locations of a simplified signaling or disease network. It must be recognized that biological systems and their malfunctions may extend beyond the scale of a few nodes, or may not be accurately approximated by such small-scale networks. In such cases, complementary strategies, such as computational approaches, might be more suitable. We will explore computational approaches to investigating the design of multiple-drug therapies in Section 6.

## 4. Mathematical Modeling of the Dynamical Aspects in Drug Combinations

In Section 2 and Section 3, we explored mathematical methods that are employed in drug combinations analysis with respect to synergism and antagonism effects. In this section, we survey other noteworthy mathematical models that capture temporal or dynamic aspects that are important in disease treatment. Specifically, the methods we examine study the dynamics of drug combinations in terms of their impact on the underlying protein concentrations associated with disease treatment, and the application of combination therapy to effectively control the evolutionary dynamics of disease states and thereby mitigate potential drug resistance.

### 4.1. Linear Relationships in Time-Varying Protein Concentrations

While drug combinations have been widely studied in the literature with respect to the pharmacodynamic outcome, Ref. [44] investigates the effect of drug combinations on protein concentrations as they vary with time following administration of one or more agents. It is important to be able to control protein concentrations using specific drug combinations to control the cell state and move from a sick state to the healthy state. The paper focuses on the protein level concentrations and employs a dynamic proteomics method [45] that evaluates the levels and locations of various proteins in living cells with respect to time. Exploring the temporal levels of expression for 15 proteins in response to several combinations of 13 drugs, Ref. [44] shows that the effect can be modeled by a weighted sum or linear superposition. The weights are time-constant but dose-dependent and describe the relative effect of every drug in combination on each protein.

Experimental results presented in [44] show that each protein has a different temporal concentration response under each drug for specific dosages. It is also revealed that the time-dependent response of a protein under the mixture of two drugs is a linear superposition of its concentration response under each of the combining drugs when considered separately. It is claimed that the weight corresponding to each drug increases by its concentration while it is also dependent on the concentration of other drugs in the combination. Therefore, if $P_{i}\left(t,c_{i}\right)$ is the protein concentration response under dose $c_{i}$ of drug *i* and $P_{j}\left(t,c_{j}\right)$ is the protein concentration response under dose $c_{j}$ of drug *j* at time *t*, we can formulate the response of the same protein under the combination of drugs *i* and *j* as:

(31) $$P_{i,j}\left(t,c_{i},c_{j}\right)=w_{i}\left(c_{i},c_{j}\right)P_{i}\left(t,c_{i}\right)+w_{j}\left(c_{j},c_{i}\right)P_{j}\left(t,c_{j}\right),$$

where $w_{i}\left(c_{i},c_{j}\right)$ and $w_{j}\left(c_{j},c_{i}\right)$ are the corresponding weights of drugs *i* and *j*, respectively. The equation proposed in [44] for weights can be formulated as $w_{i}\left(c_{i},c_{j}\right)=E_{i}\left(c_{i}\right)U_{j}\left(c_{j}\right)$, with $E_{i}\left(.\right),U_{j}\left(.\right)$ as defined in (15). However, it is mentioned in the paper that the halfway concentration of each drug depends on the concentration of the other drug in the combination. The experimental results presented in [44] show that in almost 33% of cases the weights add up to one.

Since the weights are not time-dependent, calculating (31) at two different times with similar doses of drugs (i,j) reveals the corresponding weights.

For the sake of simplicity, we drop time and dose variables from (31) and rewrite it as

(32) $$P_{i,j}=w_{i|j}P_{i}+w_{j|i}P_{j}.$$

Generalization of (31) leads to modeling the concentration response of each protein under possible combinations of the tested drugs using the data of available combination tests. For every protein and *N* different drugs with single dosages, we define $P={\left[P_{1},P_{2},\ldots ,P_{N}\right]}^{T}$ and $P_{\mathrm{II}}={\left[P_{1,2},P_{1,3},\ldots ,P_{N,N-1}\right]}^{T}$ as the vector of protein responses to single-drug and two-drug combinations, respectively. Then the matrix representation of (32) can be written as

(33) $$P_{\mathrm{II}}=WP,$$

where *W* is the weight matrix and can be stated as

(34) $$W=\left[\begin{array}{ccccc} w_{1|2} & w_{2|1} & 0 & \ldots & 0\\ w_{1|3} & 0 & w_{3|1} & \ldots & 0\\ \vdots & \ldots & \vdots & \ldots & \vdots \\ 0 & \ldots & 0 & w_{N-1|N} & w_{N|N-1} \end{array}\right].$$

Therefore, if every drug can take *m* different dosages, for *L* total proteins there are N(N−1) m${}^{2}$L unknown coefficient which should be defined.

In [44], it is shown that using the superposition formula, the response of a protein under three- and four-drug combinations can be predicted based on the protein response under two-drug combinations. They extend (31) for the response of a protein to the combination of multiple drugs and propose that the weights can be calculated based on the weights of the pairs. For the case of three drugs, the presented formulas can be expressed as following.

(35a) $$\begin{array}{cc} P_{i,j,k} & =w_{i|j,k}P_{i}+w_{j|i,k}P_{j}+w_{k|i,j}P_{k}, \end{array}$$

(35b) $$\begin{array}{cc} w_{i|j,k} & =\frac{w_{i|k}-w_{i|k}w_{j|k}}{1-w_{i|k}w_{j|k}}, \end{array}$$

where $w_{i|k}$ is the weight corresponding to drug *i* in the pair combination of (i,k). In extracting the weights relationship, the authors used the assumption that the weights add up to one, although it is not a generally valid assumption.

### 4.2. Evolutionary Dynamics and Resistance in Drug Combinations

In the case of cancer therapy, drug resistance is a frequent problem that, when it arises, returns the treatment to a former stage and leads to failure in therapeutic efforts [46]. A leading approach to combating resistance is to employ combination therapies [47]. The major reason is tumor heterogeneity, in which the tumor consists of a genetically diverse population of cells. Therefore, in every tumor there could exist a small sub-population of cells with different genetic and receptor expressions, which could cause different groups to be resistant to different drugs. Application of a single compound might therefore only be effective against a portion of the tumor population and, consequently a combination of multiple drugs is more likely to lead to a cure. Since malignant tumors are governed by evolutionary dynamics [48], evolutionary modeling is helpful for understanding drug resistance, and predicting the effects of combination therapies. Population statistics is used to model evolutionary or Darwinian dynamics, and to explain tumor heterogeneity [8]. Evolutionary modeling was shown to help to identify the cell of origin and lead to predicting the likely trends in tumor growth and drug resistance [8].

The authors in [46] present a mathematical framework for studying the evolutionary dynamics of drug resistance based on a stochastic birth-death modeling of cancerous cells. Defining $r_{g}$ and $r_{d}$ as the growth and death rates, respectively, they assume that when the size of tumor reaches $N_{t}$, which is also defined as treatment size, cancer is detected. They model the effect of a drug by introducing $r_{h}$ as the drug-induced death rate, which makes the net death rate greater than birth rate and leads to eradicating the tumor. However, mutation could happen with rate $r_{m}$ resulting in the formation of a sub-population of cells that are resistant to the drug. In the case of a single drug, in [46], Komarova and Wodarz suggest that the probability of treatment failure can be calculated as

(36) $$P_{f}=1-{\left(1-\frac{r_{h}\;r_{m}}{r_{h}+r_{d}-r_{g}}\right)}^{N_{t}}.$$

For $r_{h}$ sufficiently large, they approximate (36) to $P_{f}=1-e^{-N_{t}r_{m}}$, which is independent of growth and death rates. However, for the case of multiple drugs, the authors claim that the evolutionary dynamics of the resistance depends on the difference between growth and death rates.

The authors in [47], propose a mathematical model to predict the effects of combination therapies on eradicating the tumors. The model presented by Bozic et al. in [47] describes the evolutionary dynamics of the tumor based on a multitype branching process. The key parameters that are used in their model are the birth and death rates, the number of point mutations that potentially leads to resistance and the mutation rate. They show that, under the assumption of no cross-resistance, i.e., there are no mutations that confer resistance to more than one drug, the expected number of cells that are resistant to N-drug combinations in a tumor with size $N_{t}$ could be approximated as

(37) $$\lambda \approx N_{t}\left(\prod_{i=1}^{N}m_{i}\right)\mu^{N},$$

where $m_{i}$ is the number of point mutations that could lead to resistance to drug *i*, and $\mu =\frac{r_{m}}{s}\ln\left(N_{t}s\right)$ with $s=1-\frac{r_{d}}{r_{g}}$.

For the case of 2-drug combinations, [47] model the probability that the treatment is successful in eradicating the tumor, as the product of four independent probabilities, i.e.,

(38) $$P_{e}=p_{1}^{b}p_{1}^{a}p_{2}^{b}p_{2}^{a},$$

where $p_{1}^{b}$ is the probability that no 1-step resistant lineage arises before beginning the treatment, and $p_{1}^{a}$ is the probability that no such resistant occurs after treatment starts and during it. Analogously $p_{2}^{b}$ and $p_{2}^{a}$ are the probability of no 2-step resistant lineage arising prior and during treatment, respectively. The authors in [47] provide a closed form expression for computing these probabilities, in terms of aforementioned key parameters. The probability of treatment failure in this case, then can be calculated as $P_{f}=1-P_{e}$.

However, it worth noting that the resistance is the output of multiple random events, such as mutation, birth and death, and [47] affirms that lack of resistance in one tumor does not guarantee its absence in another identical tumor.

## 5. Discussion

**Figure 13:**
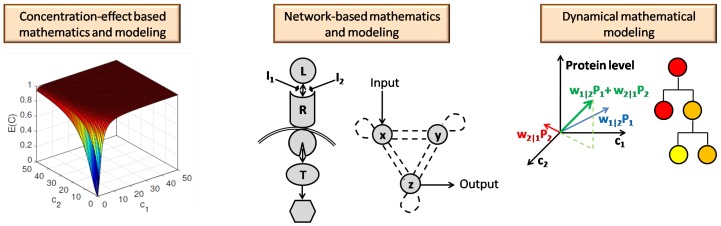
Leading mathematical methods that model drug combinations.

Figure 13 provides a pictorial summary of the mathematical methods for modeling the effects of drug combinations that have been explored in this paper. In this article, we surveyed three distinct mathematical approaches to modeling drug combinations. In Section 2, we reviewed the first group of mathematical tools, which consist of classically motivated tools that model the response to the combination therapy based on the concentration-effect relationship of each individual agent. The second group of models that we examined differed from classical methods for modeling drug combinations effects. While classical tools consider the target as a black box and do not take its underlying interaction structures into account, the second group we explored in Section 3, models the combination therapy with regards to the underlying signaling network and associated network topology of the potential targets and their relationships to each other. Analogous to the first group, these methods study the output response to the drug mixture with respect to the synergism and antagonism effects of the combination therapy. While drug combination therapies can allow for reduced overall dosages in therapeutic treatments, another benefit of combination therapies over single-drug therapies (notably in the case of cancer treatments) is their success in avoiding drug resistance. The third class of models, which we studied in Section 4, captures the evolution of underlying populations, such as concentrations of different proteins in serum or the different subpopulations associated with a tumor, during the treatment of diseases. We explored the mathematical modeling of the evolutionary dynamics, which explains drug resistance, in the third group of models in section. We investigated methods that study combination therapies through modeling their dynamical, time-varying properties. These methods differ from the classical methods, which do not reflect the effect of time in their models. From this viewpoint, it worth noting that the mathematical representation of the network-based methods, allows one to analyze both dynamic and steady-state responses, though much of the focus of their application has been on steady-state responses.

The methods that we explored in this survey may all be viewed as providing mathematical modeling tools for studying combination therapies. However, the mathematical approaches presented differ. The first group, uses the empirical pharmacodynamics concentration-response functions to compute the response of the cocktail drug. While these methods follow non-linear operations and functions, bringing up a combination modeling in a linear manner, as the method presented in Section 4.1, provides a substantial ground to apply linear mathematics and system analysis tools to generalize the model. In network-based methods, the interaction between each pair of nodes is modeled by a chemical reaction, therefore, a system of differential equations represents the whole network. Finally, all the discussed methods, perform deterministic mathematical modeling, except the evolutionary modeling that is based on stochastic random process.

**Figure 14:**
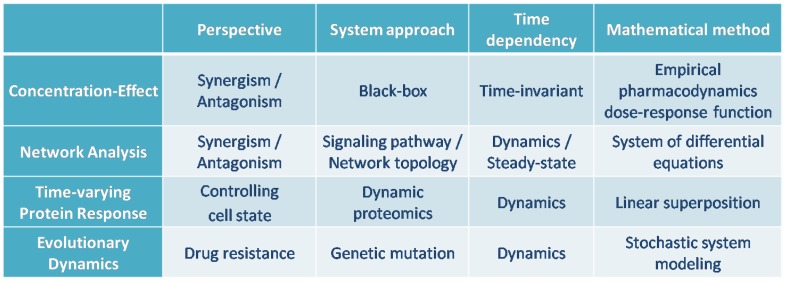
Comparison between the mathematical methods for drug combinations.

Figure 14 summarizes the comparison of the mathematical methods that were studied in this article for modeling the combination of drugs.

However, it is worth noting that different strategies have different advantages relative to each other, and further that it might be difficult to make a comparison between different strategies. For example, the notions of synergism and antagonism originated from a pharmacodynamic perspective and have subsequently been applied to capture holistic effects of a treatment. Consequently, methods such as Loewe’s and Bliss’s Independence models cannot be easily compared to models that aim to capture the time-dependent behavior of a treatment, as is considered in models that capture evolutionary dynamics.

Nevertheless, there are some limited, case by case, comparisons that often arise in the literature. As an example, we would note the use of basic network motifs allow one to capture the spectrum of synergism to antagonism in a manner that is more flexible than traditional Loewe or Bliss models can provide. Notably, traditional black-box concentration-effect models have the benefit of requiring less specific data in order to be applied, while models that include the underlying kinetics, such as network motifs would, ideally, require knowing or having data that can allow one to estimate the parameters associated with the inner workings of the model. In fact, as we discussed in Section 3, the classification of synergism versus antagonism can vary significantly with the underlying reaction coefficients and whether phosphorylation is involved.

It is our viewpoint that the choice of a model or mathematical tool should be motivated by the amount of information or data that is available or needed for the problem being considered. In fact, one of the original motivations that Loewe faced nearly a century ago was that it was beneficial to have a model that abstracted out the underlying mechanisms of action. Hence, such black-box models are beneficial in situations where the mechanisms of action or underlying kinetics are not available or unknown. Likewise, when there is information regarding the biochemical processes and the interactions involved, then network-based approaches or systems-based models become more suitable and retain a good amount of generality to explore different phenomena. Finally, as one considers a large quantity of drugs, targets, and processes (e.g., beyond pharmacodynamics, such as pharmacokinetic aspects such as absorption and metabolism of drugs), the scale of the problem increases dramatically. In such cases, a well-contained model is hard to formulate and computational tools that leverage machine learning/artificial intelligence might be the most appropriate tools to apply. Such tools typically allow one to arrive at answers to specific questions, but have less generality to explore questions beyond what they were originally intended.

## 6. Drug Combinations Based on Computational Algorithms and Machine Learning

Drug combinations have been also investigated in the literature through the application of computational methods and classification algorithms [9,49,50,51,52,53,54,55,56,57,58,59]. Computational methods can be broadly divided into algorithms that leverage a basic mathematical model at their heart but use computational algorithms for optimization, algorithms that perform a search through the potential space of combinations, and algorithms that apply statistical inference and machine learning to make predictions. In the discussion that follows, we will briefly examine these three categories.

It should be noted that there are several common challenges that computational methods face before they can be applied. For example, data sparsity is one of the main challenges in implementing computational methods since not all combinations will have been tested for different cell lines. Furthermore, a computational model that represents the combination effect of a specific set of drugs on a specific cell line cannot be easily generalized for other drugs and other cell lines [55].

### 6.1. Computational Optimization of a Mathematical Model

Every algorithm that designs optimal combinations must consider the complex dynamical network inside the cell. In [50], a computational method is provided based on integrating the ODE model for the Insulin-like Growth Factor Receptor (IGFR) and particle swarm optimization to predict the effect of targeting individual proteins on other parts of the network. The paper uses this model and experimental data to find the best combination that has minimal effects on phosphorylation of other proteins. The focus of the paper is on the cell signaling network and its kinetics, which is very challenging in terms of both modeling and acquiring experimental data. The work presented in [51] addresses the problem of intra-tumor heterogeneity and the design of treatments for cancer therapy. It is shown that some drugs that are effective in treating particular subpopulations are not even included in the optimal combination. The paper quantifies a resistance index for two subpopulations through mathematical modeling and presents an optimization problem in the form of binary integer programming for finding the optimal solution that minimizes the heterogeneous subpopulations.

Control-theoretic methods represent another class of mathematical model that has appeared in computational methods. In [9], a computational method is proposed that works with available drugs for cancer therapy. The method is based on streamlined feedback system control and leads to an approach that can support rapid experimental efforts. The work in [49] also introduces a feedback control-based method into a computational algorithm to find the optimal drug combinations for viral infections. The paper applies two cascade search algorithms, one for finding the most effective combination and the second one for decreasing the toxicity of the cocktail. The performed search algorithm, as a part of a feedback control system, is differential evolution, where the objective function is the percentage of host cells that become infected by virus.

Another category of computational approaches that employ mathematical models are those that use statistical tests to determine the relationship for how multiple drugs combine in a treatment. For example, the DIGRE approach modeled synergy sequentially, and a mathematical model was used to estimate cell death as the synergism score [60]. Goswami and Li used statistical methods to estimate a core set of genes to classify how multiple drugs affect these genes in a synergistic or antagonistic manner [60]. As another example, in [61], the authors present a Chemogenomics-based system, named ChemDIS-mixture, which is built using the previously introduced ChemDIS [62] and statistical p-tests combined with Venn diagram tools available by using the STITCH database [63]. Their approach can simultaneously analyze up to four chemical-chemical interactions with the associated interacting protein data. Two case studies, including (i) interaction between anti-tuberculosis and antiretroviral drugs, and (ii) interaction among endocrine disruptors were considered in this research.

### 6.2. Search Algorithms

Stochastic search algorithms were among the initial computational methods that implemented for the drug combinations. Finding promising drug combinations is an example of a combinatorial problem since the objective is to find a grouping of pharmaceutical agents and their dosages that satisfy criteria that indicate their potential efficacy in disease treatment. Since the potential space of drug mixtures and their doses becomes quite vast as one considers combinations from a set of many pharmaceuticals, many potential disease targets, and many dose levels, efficient strategies are needed to search these possibilities without trying each one. We briefly survey several approaches that have been presented in the literature.

A search algorithm can be viewed as an algorithm that attempts to explore a feature space (in this case, for example, the set of drugs and their dose levels) where each point in the feature space has some measure of fitness or promise. Exploring the fitness space can be viewed as exploring a terrain of hills and valleys, where the objective of the algorithm is to find the peak of one or more hills without having to try all possibilities. A further motivation behind using the search algorithms is that they do not require any information about the underlying system model. Instead, search methods typically use biomarkers for indicating the cellular responses that are fed back to the search algorithm for the next iteration.

Stochastic search algorithms are a class of search algorithms that employ randomization to help ensure that the algorithm does not halt in poor performing local maxima. Examples of stochastic search algorithms include simulated annealing, genetic algorithms, colony optimization and Gur game algorithm which have been applied to computational biology. As an example of such search methods, genetic algorithms start with an initial population of potential candidate drug combination solutions and, through a series of evolutionary steps, the population of potential solutions evolves and is measured according to a fitness criterion. Promising candidates carry on to subsequent rounds, while fewer promising candidates are excluded from further consideration. Genetic algorithms have proven promising in drug discovery [52,53]. In [52] a screening method is proposed for finding the optimal drug cocktail among an extensive number of possible combinations. The paper implements the genetic algorithm by using local search hill climbing to explore the feature space. A closed loop searching algorithm is introduced in [53] to find the optimum combination with the most potency from a large space of parameters.

The Gur game algorithm is a distributed-based stochastic search algorithm using finite state automata associated with each agent in a cooperative multi-agent system. It works based on a reward function without using a predefined model. In [54] a stochastic optimization method is proposed for finding optimal drug combinations and is compared with the Gur game algorithm. The paper claims that its method addresses two major problems of the Gur approach, which are proper normalization of drug response and proper design of a reward procedure. The work investigates how changing the concentration of a specific drug would affect the response to the combination. The presented method contains a reward function that is determined by the current and previous values of the response function when the combinations differ only in the concentration of one drug.

### 6.3. Machine Learning Methods

Another category of computational methods are those methods that employ statistical inference and machine learning methods to generate predictive models. Such algorithms learn the underlying relationship between input data from single drugs, or data involving their interactions and the effects of a limited set of drug combinations. Machine learning are an important category of computational methods since they can integrate various data types, including non-numerical data. For them to be successful, they need sufficient experimental data to estimate the statistics and inferences needed for drug combination predictions.

One broad class of machine learning algorithms are those that use labeled training data to infer the underlying relationships that can then be used to forecast drug-drug interactions for new drug-drug candidates. Labeled data typically includes sets of drugs, along with one or more labels describing the key features of those drugs and the effectiveness of that combination. Such algorithms are termed *supervised learning* algorithms, and popular supervised machine learning algorithms include algorithms that k-nearest neighbors, support vector machines (SVM), Bayesian learners, random forest, and the family of regression-based statistical classifiers [64]. We now provide a brief survey of such supervised learning algorithms have been applied to the problem of multi-drug combinations.

Bai et al. use five types of features including drugs’ targets, pathway, side effect, metabolic enzymes, and drug transporters to construct an improved Naïve Bayesian classifier that predicts the effective drug combinations [65]. Unlike the conventional Naïve Bayesian method, which assumes the probabilities of the features are independent, in this method, the distribution of the samples and the correlation between their properties are used as the prior knowledge in model construction process. The authors show that their classifier outperforms the Naïve Bayesian algorithm, SVM and K-nearest neighbor and suggest it is an appropriate classifier for biological data set with a limited sample size but a large number of properties.

The authors in [58] use machine learning algorithms to predict the combination effect of 76 pairwise combinations of 103 single drugs. They implemented SVM and Naïve Bayesian classifiers, and supplied gene expression data for a set of drugs to build their prediction models. Their work illustrated the advantage of integrating machine learning with efficient computing frameworks, such as MapReduce, to make machine learning efficient for drug effect predictions.

The authors in [56] propose a computational model to predict effective drug combinations using a machine learning-based algorithm that employs stochastic gradient boosting. Their approach uses the integration of six types of features that describe the interactions of combining drugs. The feature vectors are formed using the existence or nonexistence of a specific molecular substructure for combining drug pairs, the structural similarity between them, the similarity in protein-protein interaction networks for the drugs, the similarity score that represents the involvement of the drugs’ targets in the disease pathway, and a score that describes the probability of the chemical-chemical interaction of two agents [63]. The sixth category of features is extracted from the similarity in each level of Anatomical Therapeutic Chemical (ATC) of the drugs pair, which is defined according to the organ or system on which they affect and also based on their therapeutic, pharmacological and chemical properties [66].

Another prediction model is proposed in [59] that implements a random forest algorithm to identify promising synergistic drug combinations for cancer therapy. The authors present a feature design based on a combination of various types of features, including a similarity score for drug-target pathways, the distance between drugs’ targets in protein interaction networks, the similarity of drug chemical structures, and the specifications for differential expression of gene sets.

In [57], a machine learning method is implemented using logistic regression to predict safe drug combinations. The authors form the features required in their machine learning algorithm using data that is available describing the adverse effect for individual (single) drugs. They observe that three features contribute more than other features to the model, concluding that any drug pair containing anyone of these three features is not a safe combination.

In contrast to supervised learning algorithms, many works in the literature recognize that the data available might not be *labeled* and must rely on making inferences based on a limited set of labeled data (semi-supervised) or no labeled data (unsupervised).

Often times, the amount of labeled data available is much less than the amount of unlabeled data. Semi-supervised learning methods use the limited set of labeled data, in conjunction with various forms of mathematical constraints to make use of the unlabeled data. These constraints typically take the form of proximity constraints or that the data reside in a smaller-dimensional subspace (or manifold) of all input data. In [67], the authors used a machine learning algorithm employing manifold regularization to identify a reduced set of features that significantly affect the predictive outcome of multiple drugs being applied to cancer treatment. The resulting algorithm, the Ranking-system of Anti-Cancer Synergy (RACS), was validated on the 2014 DREAM challenge data [60], and achieved results that outperformed the best performing algorithm of the challenge, DIGRE. Another semi-supervised approach was presented in [68]. In this paper, Chen et al. used the observation that principal drugs that exhibit synergism with a similar secondary, pharmacological agent that modifies the effect of the primary agent tend to exhibit similar chemical structure, and similar drug-target interactions. The authors use the Laplacian Regularized Least Squares learning algorithm to predict promising synergistic drug combinations. Their approach used a data set consisting of 75 samples of labeled data, and over 4000 samples of unlabeled data.

In [69], the authors report the design of an algorithm that reconstructs the drug functional network based on genomic data available in the drug Connectivity Map (CMAP) database [70]. Their approach is unsupervised in the sense that their data does not use labeled data detailing prior drug combinations and their success in treatment. Instead, they use the drug functional network, in combination with the signaling network associated with a disease, to facilitate the identification of multiple drugs that collectively target a disease at many different target locations in the disease’s network. The authors report the design and theoretical underpinnings of a computational algorithm that combines the information in the disease signaling network and the targets that different drugs impact to rank the synergistic effects of drug combinations. Their results indicated a set of drugs that might be effective against lung adenocarcinoma and endocrine receptor positive breast cancer, as well as the mechanisms of action for these drug combinations. In another work, Parkkinen et al. extend the use of CMAP by applying group factor analysis. The resulting probabilistic CMAP was shown to be superior to prior methods in finding functionally and chemically similar drugs from the Connectivity Map data set [71].

## 7. Data and Software Resources

Beyond the development of models and algorithms for determining the ideal combination of multiple drugs, the design of combination therapies has a deep need for publicly available data and software resources. We now provide a brief overview of data and software resources that can support further research by the community.

Acquisition of data is essential for model determination requires, refining the inner workings of a model, estimating the underlying parameters associated with chemical equations, and building the inferences needed to make predictions. Consequently, acquisition of suitable data for analysis must be addressed before the predictions can be made. Models and algorithms that support the analysis of drug combinations typically build from a variety of different types of data, including records of previously successful drug combinations, molecular structures of pharmaceuticals, disease targets, adverse side effects, and genetic profiling of a disease.

One of the most valuable data resources for studying drug combinations are the data resources available in Connectivity Map (CMap) [70]. CMap consists of a collection of data describing how genomic expression has been altered in human cells when treated with various agents, as well as a collection of statistical algorithms that allow one to correlate genomic expression in diseases and through drug treatments. Another important database is the Drug Combination Database (DCDB) [72], which contains known examples of drug combinations collected from the Food and Drug Administration’s electronic orange book and published clinical studies. Information related to enzymes, drug transporter proteins, and drug targets is available through the DrugBank database [73]. Likewise, information associated with pathways and how these pathways are affected by drugs can be found through the KEGG database [74], while SignaLink [75] provides a more fundamental resource to analyze signaling pathway cross-talks, transcription factors, RNAs and regulatory enzymes. Information related to drug side effects can be found in the SIDER [76], FAERS [77], OFFSIDES [78] and TWOSIDES [78] databases. SIDER, for example, is a database containing descriptions of adverse side effects based on public information and marketing inserts. FAERS is a database of adverse side effects that have been reported to the FDA, and OFFSIDES is a mined version of FAERS. TWOSIDES is a database specifically targeted at relating pairs of drugs to adverse side effects based on the post-marketing surveillance data in FAERS. More detailed information related to chemical-protein interactions is available in the STITCH database [79], while protein-protein interactions are found in the HPRD [80] and DIP [81] databases. STRING is another, large database that contains information on more than 24.6 million proteins and has proven to be a useful source in studying protein-protein interaction networks [82].

In cases where existing databases are insufficient for collecting data needed to make a model or prediction, there is nevertheless the possibility that were relevant experiments previously performed and reported in the research literature. In such cases, the data must be found and supplied to the appropriate computational algorithms. Therefore, an important complementary aspect of mathematical models and data analysis is the development of approaches that can search existing collections of data that have resulted from experiments that were previously reported.

Beyond information reported to the FDA and in curated databases, data that corresponds to drug interactions are widely reported in scientific and clinical articles. Using such information presents a new hurdle to overcome transferring the data reported in an article into a database or algorithm. One approach that has been popular recently is to apply language processing algorithms [83,84] to parse the abstracts and even the main text of journal articles [85,86,87]. Other sources of data include using medical records [88], or the medical subject and substances fields contained in a journal article’s MEDLINE records. Recently reported results in [89], for example, have shown the utility of estimating drug interactions using the MEDLINE database. MEDLINE records represent an efficient summary of information contained in an article, and Lu et al. were able to show that a random sampling-based statistical algorithm that used MEDLINE records as input was able to successfully identify potential drug to drug interactions and the proteins that are likely involved in those interactions.

There are a variety of software resources available to help investigate drug combinations. An excellent survey of databases and software tools is provided in [90] for predicting synergism in drug combinations as well as network-related databases for constructing molecular interaction networks. We briefly summarize a few of them, and their key features. CompuSyn [91] is a useful free software package for quantification of Loewe-based synergism and antagonism in drug combinations. One of the drawbacks of Compusyn is that it focuses solely on the Loewe additivity model, while other tools, such as Synergyfinder [92], COMBIA [93] and Combenefit [94] incorporate additional mathematical frameworks, such as Bliss independence. It is interesting to note that Synergyfinder and COMBIA are available as a package for the R programming language. CalcuSyn [95] is another helpful tool that analyzes the effect of drug combinations and quantifies synergism and antagonism. Additionally, MacSynergy™ II [96] is a three-dimensional (3D) model to plot synergy, dose-response surfaces, and isobolograms. This analytical tool was written based on Microsoft Excel spreadsheets and macros. CAST-Compound Combination for Oncology Drug Combinations is a web-based, menu-driven system that is useful for building customized analysis tools to fit specific needs [24]. Beyond these widely available software packages, mathematical modeling languages such as R and MATLAB provide libraries that can support the modeling of biochemical networks, such as SimBiology [97]. Likewise, there are numerous tools available that support machine learning and statistical classification, such as TensorFlow [98], Torch [99] and LibSVM [100]. Since many of the problems associated with drug discovery can be computationally intensive, it is beneficial to consider computing architectures using MapReduce-based frameworks or other more general parallel-processing frameworks [101]. Many of these frameworks have standardized programming interfaces, such as Spark [102], which can facilitate the implementation of efficient search and machine learning algorithms. Such tools promise to expand the capabilities reported for Hadoop-based drug discovery reported in [58].

## 8. Conclusions and Discussion

Therapies that involve the use of multiple pharmaceuticals often have superior effects compared to treatments involving a single agent. To take advantage of the potential benefits of multiple drugs in treatment, it is necessary to have improved understanding of how the effects of multiple agents combine. In this review, we have explored mathematical methods that model combination therapies from various perspectives. We investigated methods that predict the synergism and antagonism effects of the combination through classic black-box models, such as the well-known Loewe or Bliss models. We also examined methods that aim to capture the underlying signaling networks and the topology of the drug targets and their relationships. We also reviewed evolutionary modeling of combination therapies as an effective solution to combating drug resistance in cancer treatment. Regardless of whether the mathematical method is dynamic or time-invariant, presents non-linear or linear combination, relies on deterministic or stochastic methodologies, the final goal is to find the optimum combination with maximum effectiveness and minimum toxicity. Additionally, in this article, we explored computational algorithms, including machine learning and statistical search algorithms that analyze data to predict promising drug combinations. We also provided a review of some helpful tools and software in combination therapy and interaction networks, as well as references to some valuable data resources.

There are various challenges that arise while constructing the model, analyzing data, evaluating the model and performing in vitro and in vivo assays for combination therapy. Although modeling and experimental efforts have proposed various efficient combination therapies, many of them fail to be approved by the FDA or other agencies for clinical usage. One reason is that the molecular and genomic profiles that have been used in model construction might be different from the actual profiles of actual patients. Additionally, models are often constructed using sparse data sets and only a few specific cell lines, and thus cannot be easily and practically generalized, making the case for supporting clinical trials more difficult. Likewise, not all the features and characteristics of the combining drugs might have been understood and considered while constructing the computational model. Therefore, moving from mathematical/computational models and pre-clinical assays to clinical combination trials requires consideration of many practical factors. Clinical combination therapy requires precise designs and should inform pre-clinical studies since, otherwise, empirical clinical experiences could take a prohibitive amount of time. Another challenge that arises in the field of drug combinations is that most of the modeling and experimental investigation is based on an ideal behavior for one or more drugs. In actuality, however, drug delivery mechanisms are an important aspect that modulates the behavior of a pharmaceutical and consequently how the combination is delivered could dramatically affect the costs and outcomes. Likewise, the impact of pharmacokinetic aspects related to a drug’s lifetime in a patient also affects the efficacy of a medical treatment, and whether the full range of therapeutic (and, unfortunately, also the toxic) effects are probable. Therefore, models and computational algorithms for multiple-drug therapies should strive to capture the full lifetime of a treatment, from drug delivery to absorption, distribution, metabolism, and excretion of the compounds. The lack of negative data sets could be counted as another challenge in computational modeling since successful drug combinations tend to be reported and marketed, while it is much less common for negative combinations to be marketed or reported.

Looking forward to future developments in modeling drug combination therapies, it should be noted that future research will be needed to be able to quantify the unique, patient-specific properties that parameterize the different drug combinations models. Being able to accurately estimate variations in different patients’ synergistic effects, or variations of rates associated with the underlying chemical reaction equations, or the different subpopulations that might exist in a patient will be essential to driving the application of drug combinations models for personalized therapies.
